# A Regional Survey of Attitudes and Experiences in Determining When to Prescribe Intranasal Naloxone for Patients Seen in Palliative Care Clinics in the United States of America

**DOI:** 10.1089/pmr.2024.0027

**Published:** 2024-07-19

**Authors:** Sean Marks, Divya Patel, Rabia Amjad, April Dawson, Rebekah J. Walker, Drew A. Rosielle

**Affiliations:** ^1^ Medical College of Wisconsin, Milwaukee, Wisconsin, USA.; ^2^ Peace Health Southwest Medical Center, Vancouver, Washington, USA.; ^3^ University of Minnesota Medical Center, Minneapolis, Minnesota, USA.

**Keywords:** clinician survey, naloxone, opioid overdose, outpatient naloxone, palliative care

## Abstract

**Background:**

Published guidelines that help clinicians identify patients who would benefit from the co-prescription of intranasal naloxone (IN) exclude “palliative care patients.” In the absence of clear care standards, palliative care (PC) clinicians may experience uncertainty in how to approach IN co-prescriptions.

**Objective:**

Explore the attitudes of PC clinicians in the United States of America who work at regional health care institutions regarding IN prescriptions for patients they prescribe opioids for.

**Methods:**

An 18-question electronic survey was distributed to PC clinicians that practice at institutions in Wisconsin or Minnesota with at least 10 other PC clinicians between February and May 2023. The survey explored clinical scenarios in which respondents would and would not prescribe IN.

**Results:**

Fifty-six PC clinicians responded to the survey—response rate 41%. Most respondents (90.9%) did not feel IN prescriptions should be reserved for patients with a full code status; 67.9% of respondents felt that IN prescriptions are reasonable for certain patients with a terminal illness and comfort goals of care. Neither prognosis, duration of opioid therapy, nor dose of opioid therapy were significant factors in determining whether most respondents prescribed IN for their patients. Most respondents (81.8%) felt clinician counseling and patient consent were essential before prescribing IN.

**Conclusion:**

Most PC clinicians in our survey felt that IN prescriptions can be appropriate for patients they prescribe opioids for. Bystander safety was an emerging rationale for why respondents chose to prescribe IN for their patients. Despite public health efforts to make IN more freely available, most respondents felt clinician counseling was essential before prescribing IN for their patients.

## Introduction

Opioids are commonly prescribed for analgesia and dyspnea relief.^1^ Respiratory depression, sedation, and overdose are recognized adverse effects from prescribed opioids.^2^ Safe-opioid-prescribing guidelines, such as those from the Centers for Disease Control (CDC) and Veterans Affairs (VA), encourage clinicians to co-prescribe intranasal naloxone (IN) as a potential antidote for a potentially fatal opioid overdose for patients regularly prescribed opioids for chronic, nonmalignant pain control.^3,4^

However, safe-opioid-prescribing-guidelines from the CDC do not pertain to patients “receiving palliative or end-of-life care”^3^ and VA guidelines suggest against prescribing naloxone for patients receiving “palliative care.”^4^ While it can be difficult to define patients who are receiving “palliative” or “end-of-life” care, many patients seen by palliative care (PC) teams are receiving disease-modifying therapies, have life-prolonging goals of care, and prognoses of many years.^5,6^ It is reasonable to assume then that some PC clinicians think patients they prescribe opioids for would benefit from a co-prescription of IN to reduce the chances of an opioid overdose for either the patient or a bystander, even if available guidelines do not explicitly encourage them to do so routinely.^7^ Conversely, there could be unintended negative consequences of IN administrations for patients who are terminally ill.^8^ Beyond cost concerns of the medication, distressed family members may provide IN to an imminently dying patient receiving comfort-focused, home hospice care. This could precipitate an uncomfortable crisis in the hours or days before death via acute opioid withdrawal.^4,9^

In the absence of clear direction from published guidelines for prescribing IN for patients receiving palliative or hospice care, PC clinicians may feel uncertain regarding the clinical appropriateness of IN for patients they are caring for.^10^ The purpose of this study was to obtain background survey data on the attitudes and experiences of regional PC clinicians who work at large health care institutions in Wisconsin and Minnesota regarding IN prescriptions for their patient population. By doing so, we hope to identify where there is and is not consensus among PC clinicians for prescribing IN. Our hope is that the results could eventually help establish standards of care for the co-prescription of IN naloxone for patients seen by PC clinicians.

## Methods

We reviewed the published literature on IN in patients receiving palliative or end-of-life care and informally examined the care tensions in clinical practice at our PC team meetings to identify potential gaps in knowledge and practice. The authors then designed an electronic (Qualtrics^®^), anonymous survey with the goal of attaining a better understanding of what patient care scenarios PC and/or hospice care clinicians would deem IN prescriptions to be appropriate. The survey was 18 questions included yes/no and multiple-choice questions regarding demographics (*n* = 4) and clinician attitudes/experiences regarding IN co-prescriptions based on a variety of care considerations including (1) prognosis, (2) patient care preferences, (3) opioid prescribing factors, (4) level of concern regarding opioid overdose, and (5) recommended patient counseling when prescribing IN. Five of the questions provided a blank comment box for respondents to offer corresponding commentary to their multiple answer choice. The study was reviewed and approved by the Medical College of Wisconsin Institutional Review Board (PRO00042965). It was a voluntary, open survey for which no monetary reward was provided to those who completed it.

### Participants

We identified PC clinical leaders at three academic centers in Wisconsin and two academic centers in Minnesota with at least 10 PC clinicians on their team. The PC leaders were asked via electronic mail to forward the link to the survey along with an electronic letter describing the voluntary survey to all prescribing clinicians within their team who have trained and/or currently practicing as a palliative or hospice care provider. The invitation to participate included informed consent language informing participants that the survey would take about 5–10 minutes to complete, there was no material benefit for them to participate in the survey other than helping the study results, and that the survey was anonymous. Exclusion criteria were clinicians who are not trained or practicing in hospice or PC in Minnesota or Wisconsin.

### Study procedures

The electronic survey was conducted over eight weeks from February to April 2023 using an initial invitation letter and two follow-up letters sent to the institutional leaders via electronic mail with instructions to forward the reminder letters to potential participants on their team.

### Statistical analysis

Summary statistics were run to describe the study participant characteristics and summarize responses to questions. To investigate differences between physicians and nonphysicians, we computed descriptive statistics using frequency and percentages for categorical variables stratified by type. Next, chi-square or Fisher’s exact test was performed. In addition, descriptive statistics were conducted to investigate differences by region (Wisconsin, Minnesota). Again, chi-square or Fisher’s exact tests were used to compare frequency and percentages. Statistical significance was set at *p* value <0.1. Analyses were performed using SAS 9.4 (SAS Institute, Cary NC).

## Results

One hundred and thirty-three clinicians received the e-mail invitation to complete the survey of which 56 responded to at least one question, yielding a response rate of 42.1%. Forty-seven of the clinicians who received an invitation to participate in the survey were from Minnesota (35 from institution A; 12 from institution B); 86 were from Wisconsin (27 from institution C; 23 from institution D; 36 from institution E). Of the 56 respondents, 23 were from Minnesota, 33 were from Wisconsin. See Table 1 for baseline characteristics of study participants.

**Table 1.** Baseline Characteristics of Study Participants (*n* = 56)

Question	% of respondents (*n*)
Type of medical provider	
Physician	51.8 (29)
Physician assistant	3.6 (2)
Nurse practitioner	39. (22)
Pharm D	0
Clinical nurse specialist	5.4 (3)
Other	0
Predominant patient population of palliative care (PC) practice	
Adult	91.1 (51)
Pediatric	8.9 (5)
In which setting are you seeing palliative or end-of-life patients as part of your clinical practice (Select all that apply)	
Outpatient PC	41.1 (23)
Inpatient PC consults	85.7 (48)
Home PC	5.4 (3)
Inpatient/residential hospice	23.2 (13)
Home hospice	16.1 (9)
Other	7.1 (4)
State of PC or end-of-life care practice	
Minnesota	41.1 (23)
Wisconsin	58.9 (33)

### Respondents’ attitudes and experiences regarding prognosis

When asked, *“When considering IN naloxone, does prognosis factor into your clinical decision-making on whether you prescribe it or not?”* all 56 respondents responded, 25.0% (*n* = 14) replied yes; 75.0% (*n* = 42) responded no. Two respondents entered free text comments suggesting that goals of care and patient care preferences have more of a factor than prognosis in whether they prescribe IN. When asked, *“In general, how limited do you think the expected prognosis should be before you would routinely recommend AGAINST any IN naloxone prescribing?”* 42 of 56 responded to the question, 79.6% (*n* = 39) choose answer choice <2 weeks; 30.6% (*n* = 15) chose weeks to 3 months; 6.1% (*n* = 3) chose 3–6 months, 4.1% (*n* = 2) chose 6–12 months; and 4.1% chose >12 months.

### Respondents’ attitudes regarding patient care preferences

Fifty of 55 (90.9%) total respondents answered “No” to the survey question *“In general do you think IN naloxone prescriptions should be reserved for patients who are full code.”* Concern for the potential overdose of bystanders and concern for treating “full code” patients differently were mentioned as free text reasoning for why they selected “No”. Thirty-eight (67.9%) of 56 respondents answered “Yes” when asked if they would find it reasonable to prescribe IN to a patient with a terminal illness and goals of care for comfort-only focused treatments (e.g., a patient receiving home hospice care). Seventy-five percent of 40 respondents felt that having high-risk patients or bystanders in the home along with a prolonged prognosis were both reasonable justifications for prescribing IN for PC patients with comfort goals of care. One respondent expressed via free text they would not ever prescribe IN for a patient receiving comfort-focused care because they felt it would be too confusing for families to determine when to administer it since sedation is typically a given in the dying process.

### Respondents’ attitudes regarding opioid-related factors for choosing whether to prescribe IN

**FIG. 1.**
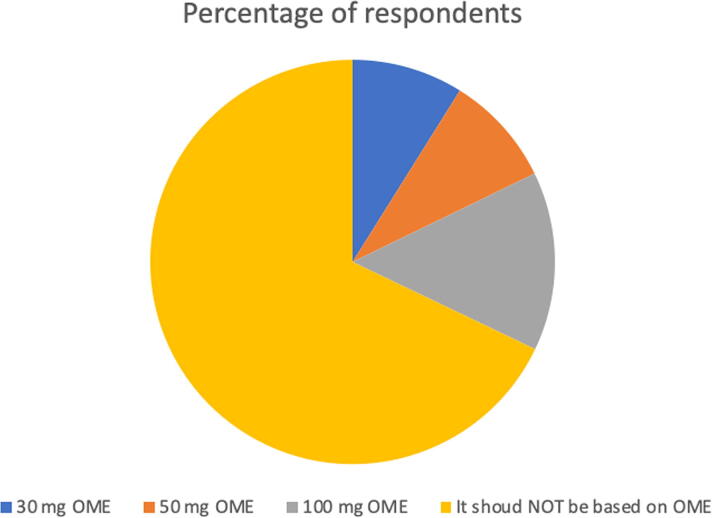
“Assume your patient had no extra risk factors for being harmed by long-term opioid therapy. Which daily oral morphine equivalent (OME) dose cutoff (meaning the lowest possible dose that should trigger co-prescription of IN) do you think is most appropriate when deciding when to co-prescribe IN naloxone?” Total number of responses: 56.

The duration of anticipated opioid therapy was not considered to be a significant factor in deciding whether to prescribe IN for 43 of 55 respondents (78.2%). In a follow-up question, 15 of 17 respondents (88.2%) chose >3 months as a minimum duration of opioid therapy in which they would prescribe IN naloxone, 8 (47.1%) chose 1–3 months; and 6 (35.3%) chose <1 month. Thirty-six of 56 respondents responded that they would be more likely to prescribe IN for patients being prescribed both opioids and benzodiazepines. See Figure 1 for respondents’ attitudes regarding daily oral morphine equivalent (OME) dose cutoff and IN prescribing.

### Respondents’ attitudes and experiences regarding opioid overdose

**FIG. 2.**
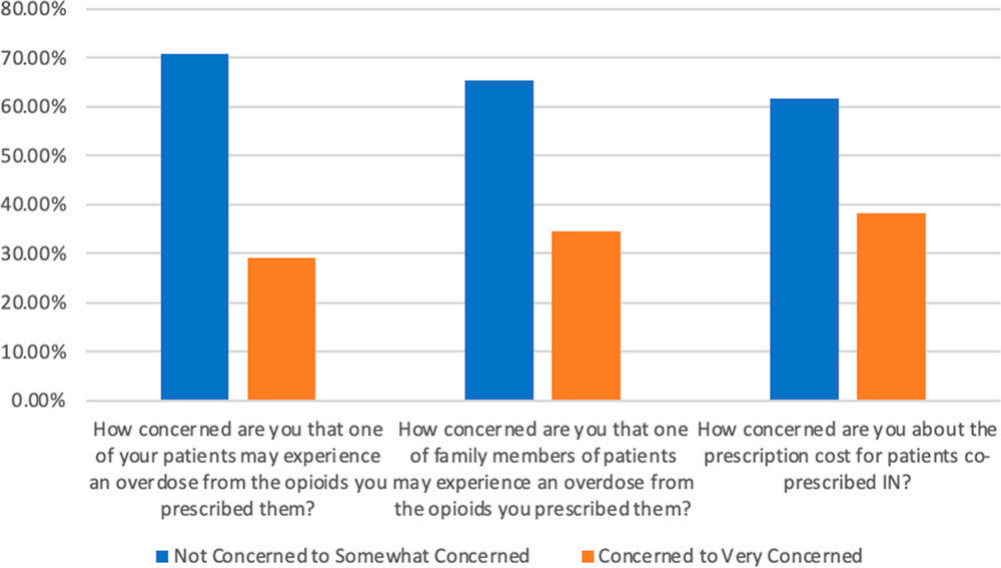
Respondents’ level of concern for patient opioid overdose, bystander opioid overdose, and prescription cost of IN. Number of respondents: 55 for each question.

Thirty-six of 56 respondents (64.3%) responded that they had never had a patient who had a serious opioid overdose from opioids they prescribed to them. Of the 19 who responded “Yes,” 9 responded that the overdose led to IN administration but nothing worse; 8 responded it led to hospitalization/emergency room visit; 2 responded that it led to death. See Figure 2 for the results of survey questions assessing respondents’ level of concern regarding opioid overdose and prescription cost.

### Respondents’ attitudes regarding essential patient counseling for IN prescribing

**FIG. 3.**
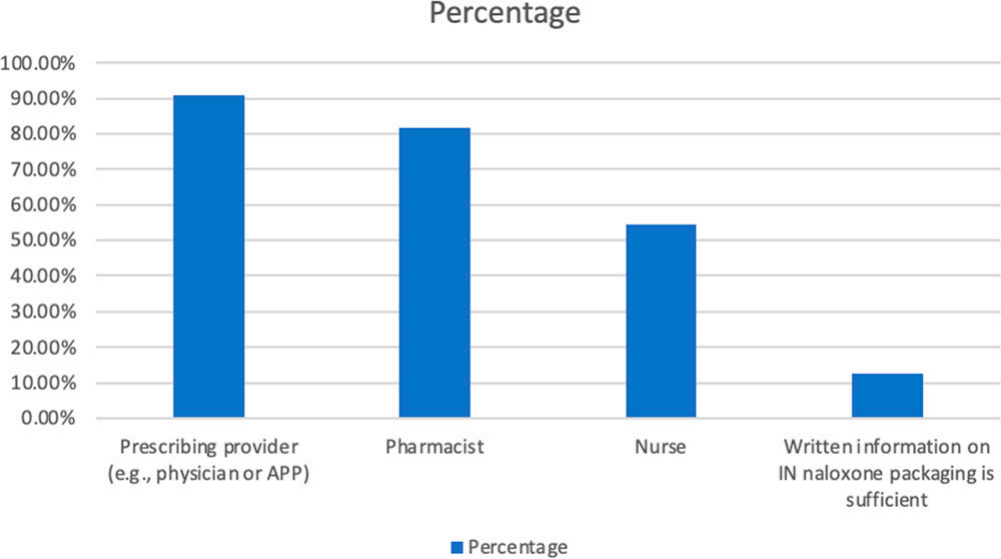
Who do you think should provide the patient education counseling about IN naloxone when is prescribed? Select all that apply (*n* = 55 respondents).

Three questions assessed respondents’ attitudes regarding how best to counsel patients when IN is prescribed. Forty-five of 56 (81.8%) respondents answered the “Yes” regarding whether they feel it is essential for clinicians to counsel patients and receive their consent before prescribing IN. When asked *“Do you think clinician counseling can be a barrier to IN naloxone prescribing?”* 73.4% of 55 respondents answered no. See Figure 3 for results regarding which health care providers survey participants feel should be providing the patient counseling for IN.

### Comparisons by clinician type and region

When physician versus nonphysician clinician responses were compared, there was no statistically significant differences in how these groups answered key questions regarding how prognosis, patient care preferences, and opioid prescribing factors affect their IN prescribing patterns. Additionally, there were no statistically significant differences in their concern for opioid overdose, their attitudes regarding IN patient counseling, and their state of practice. When investigating differences by state, 24.2% of 33 Wisconsin respondents (*n* = 8) answered that a patient they prescribed opioids for had experienced an opioid overdose compared with 43.5% of the 23 Minnesota respondents (*n* = 10). However, there were no statistically significant differences in how respondents from the two states answered key questions.

## Discussion

Our survey findings suggest that many Wisconsin and Minnesota-based PC clinicians feel that IN prescriptions are appropriate to whom they are prescribing opioids. In fact, our survey found that most respondents thought it was appropriate to prescribe IN not just for patients with life-prolonging goals of care, but for patients with comfort-focused goals of care or specific treatment limitations established such as “do not resuscitate” or “do not intubate” orders. Having a prognosis greater than 2 weeks and/or a high-risk patient, family member, or home setting were identified as patient factors that would compel our respondents to prescribe IN for patients with comfort-focused goals of care. This suggests that many practicing PC providers in our region do not feel their patients should be excluded from IN prescriptions as recommended in CDC and VA guidelines.^3,4^

Opioid-prescribing factors did not appear to have a strong effect on how our respondents determine whether to prescribe IN for their patient population. Nearly 80% of respondents felt that duration of opioid therapy did not matter in this patient care decision and 67.9% did not feel the decision to prescribe IN should be based on OME dose cutoffs. This suggests that some electronic medical record (EMR) prescribing prompts, which encourage clinicians to prescribe IN if over certain OME doses, if duration of opioid therapy is longer that 3 months, and/or benzodiazepines are being co-prescribed^11^ may not align with the attitudes and clinical opinions of the regional PC clinicians we surveyed. While 64.3% of respondents stated they would be more likely to prescribe IN if their patient was also on benzodiazepine therapy, we wonder if this patient care factor may be overthinking the issue of IN appropriateness too. Considering that some respondents in our survey have had patients they cared for who have experienced significant and potentially preventable harm from opioids via overdose and considering the reasonable concern our respondents had for the health of bystanders in the patient home, we feel it would be reasonable for EMR tools to trigger prompts for the co-prescription of IN for any patient being prescribed opioids regardless of OME dose, PC status, duration of opioid therapy, or co-prescription of benzodiazepines, as long as clinicians had the ability to opt out of the IN co-prescription if they were concerned that the patient could be at risk of imminent death and IN administration could negatively impact their comfort level during their dying process.

The survey results regarding patient counseling and informed consent for IN prescriptions surprised us. Since the time of the survey in the early spring of 2023, IN has become more widely adapted throughout the United States and elsewhere. This has included the implementation of public health vending machines^12,13^ and public facing campaigns such as the National Harm Reduction Coalition’s Naloxone Finder that provide easily accessible information to patients on where to find free naloxone.^14^ So, it is possible that our respondents’ attitudes and experiences regarding essential patient counseling for IN prescriptions has evolved since the spring of 2023. Regardless, we were not expecting that most respondents would feel that patient counseling and an informed consent process from a prescribing clinician would be considered as an essential component of an IN-prescription order. In fact, 81.8% of respondents felt it was essential for a prescribing clinician (physician or advance practice provider) to counsel a patient about IN and receive their consent before prescribing it. Furthermore, only 12.7% respondents felt that the written information on IN packaging was sufficient patient education to prescribe IN. As IN becomes more widely and freely available, we are curious to see how PC clinician attitudes regarding essential patient counseling evolves for IN prescriptions.

Our survey study has many limitations. It is a small sample size of 56 respondents, all in the same region, and with a response rate of 42.1% that is similar to other web-based clinician surveys.^15^ While the lack of regional variation was a purposeful methodologic design (we specifically aimed to see if there was a regional standard of care regarding IN prescribing which if known may reduce some of the perceived uncertainty that accompanies this care decision by regional PC providers), there may be an inherent selection bias among our respondents compared with the general pool of PC clinicians. Our survey is not a validated survey instrument; hence it is likely that our language choice may have created lead bias. It was an open survey, so it is possible that some respondents completed the survey more than once. Finally, we regret not inquiring about counter-balancing factors to IN prescriptions. Particularly, whether respondents experienced any patients under their care who have been harmed by IN prescriptions they provided as a PC clinician.

## Conclusion

Most PC clinicians who responded to our survey expressed that IN prescription can be an appropriate order for terminally ill patients receiving palliative or end-of-life care. Concern for opioid overdose of not just the patient, but patient bystanders was a compelling reason to prescribe IN for their patient population.

## Abbreviations Used

APP: advanced practice provider
EMR: electronic medical record
IN: intranasal naloxone
OME: oral morphine equivalent
PC: palliative care
VA: Veterans Affairs
